# High Interlaboratory Reproducibility and Accuracy of Next-Generation-Sequencing-Based Bacterial Genotyping in a Ring Trial

**DOI:** 10.1128/JCM.02242-16

**Published:** 2017-02-22

**Authors:** Alexander Mellmann, Paal Skytt Andersen, Stefan Bletz, Alexander W. Friedrich, Thomas A. Kohl, Berit Lilje, Stefan Niemann, Karola Prior, John W. Rossen, Dag Harmsen

**Affiliations:** a Institute of Hygiene, University Hospital Münster, Münster, Germany; b Department of Microbiology and Infection Control, Statens Serum Institut (SSI), Copenhagen, Denmark; c Department of Medical Microbiology, University of Groningen, University Medical Center Groningen, Groningen, The Netherlands; d Molecular and Experimental Mycobacteriology, Forschungszentrum Borstel, Leibniz-Zentrum für Medizin und Biowissenschaften, Borstel, Germany; e German Center for Infection Research (DZIF), Borstel, Germany; f Department of Periodontology and Restorative Dentistry, University Hospital Münster, Münster, Germany; Medical College of Wisconsin

**Keywords:** whole-genome sequencing, ring trial, interlaboratory reproducibility, cgMLST, molecular subtyping

## Abstract

Today, next-generation whole-genome sequencing (WGS) is increasingly used to determine the genetic relationships of bacteria on a nearly whole-genome level for infection control purposes and molecular surveillance. Here, we conducted a multicenter ring trial comprising five laboratories to determine the reproducibility and accuracy of WGS-based typing. The participating laboratories sequenced 20 blind-coded Staphylococcus aureus DNA samples using 250-bp paired-end chemistry for library preparation in a single sequencing run on an Illumina MiSeq sequencer. The run acceptance criteria were sequencing outputs >5.6 Gb and Q30 read quality scores of >75%. Subsequently, spa typing, multilocus sequence typing (MLST), ribosomal MLST, and core genome MLST (cgMLST) were performed by the participants. Moreover, discrepancies in cgMLST target sequences in comparisons with the included and also published sequence of the quality control strain ATCC 25923 were resolved using Sanger sequencing. All five laboratories fulfilled the run acceptance criteria in a single sequencing run without any repetition. Of the 400 total possible typing results, 394 of the reported spa types, sequence types (STs), ribosomal STs (rSTs), and cgMLST cluster types were correct and identical among all laboratories; only six typing results were missing. An analysis of cgMLST allelic profiles corroborated this high reproducibility; only 3 of 183,927 (0.0016%) cgMLST allele calls were wrong. Sanger sequencing confirmed all 12 discrepancies of the ring trial results in comparison with the published sequence of ATCC 25923. In summary, this ring trial demonstrated the high reproducibility and accuracy of current next-generation sequencing-based bacterial typing for molecular surveillance when done with nearly completely locked-down methods.

## INTRODUCTION

Today, next-generation sequencing (NGS) is increasingly used to determine the genetic relationships of bacteria on a nearly whole-genome level for infection control purposes and phylogenetic studies. In a shotgun approach, fragmented bacterial DNA is usually sequenced in a highly parallel way, resulting in millions of short reads (up to 400 nucleotides in length) that are either compared to an ideally closely related reference genome (mapping) or are assembled *de novo* for the subsequent extraction of genomic information. Currently, two different approaches, based on single nucleotide polymorphisms (SNPs) (1, 2) or allelic changes (core genome multilocus sequence typing [cgMLST]) (3–5), are used to extract whole-genome sequencing (WGS) information for subsequently displaying the genotypic relationship.

For continuous infection control surveillance, typing methods should be highly reproducible, ideally generating identical typing results across different laboratories. Previously, we demonstrated that this is the case for spa typing that is based on the DNA sequence determination of a repetitive region of the protein A gene (*spa*) of Staphylococcus aureus using Sanger sequencing (6). For NGS data, it is known that different sequencing technologies exhibit different error characteristics at the read level (7, 8). Moreover, the analysis pipelines, including assemblers and analytical parameters, can influence the final typing results (7, 9, 10). However, it is unknown how reproducible the overall process of WGS-based bacterial typing is when applied in a multicenter study.

Therefore, we investigated the reproducibility and accuracy of microbial WGS-based typing, employing an international ring trial of five laboratories in three European countries (Denmark, Germany, and The Netherlands).

## RESULTS AND DISCUSSION

All five laboratories met the minimum run quality criteria in a single run without repetition (Table 1). Mean sample coverage was 131-fold. However, the coverage per sample varied markedly between 29- and 256-fold, but only samples NGSRT07C1 and NGSRT16C3 exhibited coverages of <75-fold (see Table S1 in the supplemental material). Also, the mean N50 assembly metric parameters differed markedly, whereas the mean percentages of called cgMLST targets were quite even between the laboratories (Table 1). Sample N50 values and percentages of called cgMLST targets were consistently low in samples with <75-fold coverage (see Table S1). All of the reported spa types, sequence types (STs), ribosomal STs (rSTs), and cluster types (CTs) were identical (see Table S1). Also, Sanger sequencing-based spa typing and BIGSdb revealed identical spa types, STs, and rSTs. Only in the two low-coverage samples, the rST, CT, and also the ST for NGSRT16C3, were not assigned. Moreover, in sample NGSRT13C3, the sequence of the 16 repeats containing spa type t032 was not determined.

**TABLE 1:** Summary of sequencing run characteristics and cumulative analysis results from the five participating laboratories

Laboratory designation	Sequencing run characteristics					Sequencing analysis results	
	Cluster density (K/mm^2^)	Run output (Gb)	% reads >Q30	Mean read length (bp)^*a*^	Mean fold coverage (SD^*b*^, range)^*c*^	Mean N50	Mean % called cgMLST targets (SD, range)
C1	935	8.6	89.9	225	129 (27, 43–170)	56,620	97.5 (5.7, 73.3–99.6)
C2	715	6.7	92.2	237	106 (17, 76–144)	153,228	99.2 (0.4, 98.0–99.8)
C3	1,297	10.6	87.7	238	169 (54, 29–256)	253,745	98.9 (2.2, 89.7–100)
C4	878	8.2	88.4	221	123 (16, 98–164)	101,873	99.2 (0.4, 98.5–99.9)
C5	1,247	10.8	78.1	180	129 (24, 86–187)	225,594	99.3 (0.3, 98.7–100)
Total mean	1,014	9.0	87.3	220	131 (27, 66–184)	158,212	98.8 (1.8, 91.6–99.9)

a Read lengths after Illumina base calling and adapter removal.

b SD, standard deviation.

c Coverage is calculated for an S. aureus genome size of 2.8 Mb.

**FIG 1:**
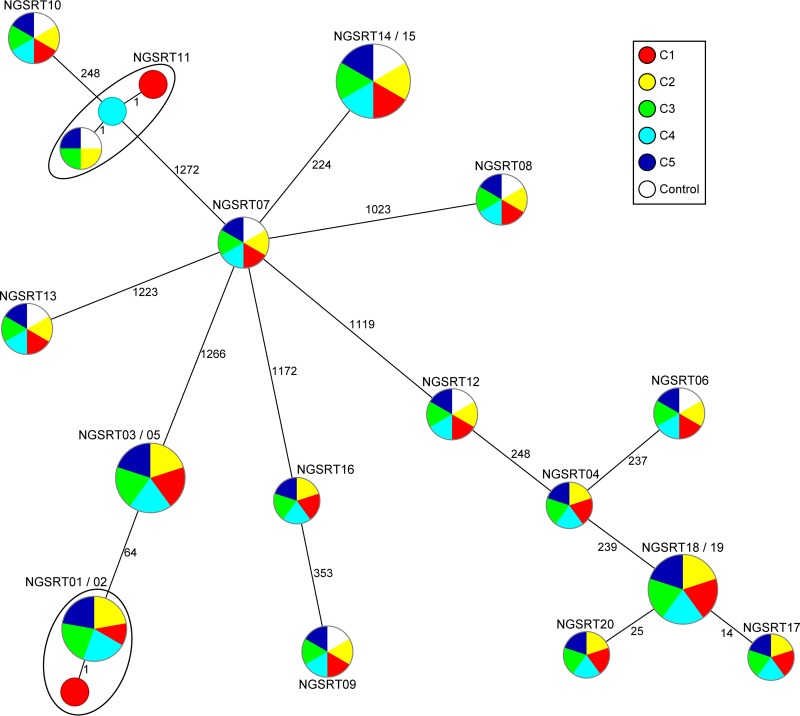
Minimum-spanning tree illustrating the comparison of cgMLST results from the 20 S. aureus isolates sent to five laboratories (C1 to C5) in a blinded fashion. Each circle represents a single genotype, i.e., an allelic profile based on up to 1,861 target genes (23) present in the isolates with the “pairwise ignoring missing values” option turned on in the SeqSphere^+^ software during comparison. The circles are named with the sample ID(s) colored by the participating laboratory, and the sizes are proportional to the number of isolates with an identical genotype. The numbers on connecting lines display the number of differing alleles between the connected genotypes. The control samples colored in white originated from independent cultivations and DNA extractions of samples NGSRT06 to NGSRT15.

In-depth analysis of the up to 1,861 reported cgMLST genes per sample demonstrated that the majority of isolates shared identical allelic profiles (Fig. 1). A comparison with the controls (NGSRT06-15), which exhibited no deviation, further corroborated this high reproducibility independent from DNA extraction. Samples NGSRT11C1 and NGSRT11C4 varied in one gene (hypothetical protein, SACOL0424), very likely due to a misassembly at the end of the gene. Also, for NGSRT02C1, a wrong allele was called in SACOL2642 (hypothetical protein) due to a low local coverage of 2-fold. These findings are in line with those of a previous study, where an N50 plateau effect for Illumina data was noted above a threshold of 75-fold average coverage (7).

In total, of 183,927 cgMLST allele calls, only 3 (0.0016%) were wrong, resulting in an average of 0.03 wrong alleles per sample. This low error rate does not significantly affect the ruling in or out of samples during outbreak investigations (11) and ensures high intra- and interlaboratory reproducibilities.

To control the accuracy, the 1,861 cgMLST target sequencing results from sample NGSRT16, i.e., ATCC 25923, which were identical for all five laboratories (Fig. 1), were compared with the published genome sequence of this strain (NZ_CP009361) revealing 12 single nucleotide polymorphisms (SNPs) (see Table S2). Suitable primers for amplifying and subsequent Sanger sequencing were designed for the 12 regions spanning these SNPs (see Table S2) and confirmed that all 12 SNPs were correctly determined during the ring trial. Most likely, the discrepancy with the published sequence can be explained by microevolutionary events that occurred during the freezing, thawing, and repeated cultivation of this strain, which was originally isolated from a clinical specimen in 1945 (12). Similar effects were already previously detected (7).

Our study comprises four limitations. First, we sent only DNA instead of living organisms to the five laboratories, and thereby did not test the influence of DNA extraction methods. The results from the 10 controls analyzed in parallel in the ring trial organizer's laboratory indicated that this might be of minor importance, as long as high-quality DNA is deployed. Second, we only compared data from one type of sequencing machine and one type of sequencing chemistry. However, there is evidence from the literature that results from a single laboratory are not significantly biased by the sequencing machine nor by the sequencing chemistry (13, 14). Third, all of the ring trial participants used the same software for analysis. To partially address this issue, we used another tool to verify ST and rST assignment and demonstrate reproducibility across different tools. The Global Microbial Identifier (GMI) wet- and dry-laboratory proficiency test attempts to overcome such limitations, but is challenged by making in-depth comparisons of the heterogeneous results (15). Finally, the accuracy was only determined for cgMLST targets. In accordance with recent practices in public health and clinical microbiology (16), the intergenic regions in particular were not controlled here.

In summary, with the shown high reproducibility and accuracy of WGS-based microbial typing when using a standardized methodology, our study provides the basis for a proficiency testing program, which is one crucial component for ensuring the quality of next-generation sequencing in clinical laboratory practice (17).

## MATERIALS AND METHODS

Twenty Staphylococcus aureus DNA samples (NGSRT01 to NGSRT20) (Table 2), selected from a diverse collection of isolates (livestock-associated, community-/hospital-acquired methicillin-susceptible and -resistant S. aureus from sporadic cases and outbreaks, and a quality control strain), along with duplicates to assess intralaboratory reproducibility, were distributed in a blind-coded manner to the five participating laboratories. DNA samples were prepared using the MagAttract HMW DNA kit (Qiagen, Hilden, Germany) in accordance with the manufacturer's instructions with the addition of 120 U lysostaphin (Sigma, Taufkirchen, Germany) to lyse methicillin-resistant S. aureus (MRSA). In addition, the laboratories received a protocol (supplemental material) for performing a single sequencing run on an Illumina MiSeq sequencer using the Nextera XT library preparation kit and the 250-bp paired-end sequencing chemistry version 2 (Illumina, San Diego, CA, USA). Sequencing indices from the Nextera XT index kit were used for multiplexing; participants were free to choose any index combination for the samples. The run acceptance criteria were a sequencing output >5.6 Gb (to achieve an average sequencing coverage of >100-fold for the 20 samples with genome sizes of 2.8 Mb) and a Q30 read quality score of >75%. Otherwise, the sequencing run had to be repeated. SeqSphere^+^ software version 2.4 or higher (Ridom GmbH, Münster, Germany) run on a Microsoft Windows operating system was used with default parameters for quality trimming, *de novo* assembly, and allele calling. Specifically, reads were trimmed at their 5′- and 3′-ends until an average base quality of 30 was reached in a window of 20 bases and subsequently down-sampled to 120-fold coverage. *De novo* assembly was performed using the incorporated Velvet tool version 1.1.04 and a SeqSphere^+^ specific k-mer optimization procedure (18). SeqSphere^+^ searched the defined genes using BLAST (19) with parameters described previously (20). In addition, the genes were assessed for quality, i.e., the absence of frameshifts and ambiguous nucleotides. A gene was called only if all above-mentioned criteria were met. Thus, determined spa types (10), MLST sequence types (ST) (21), ribosomal MLST types (rST) (22), cgMLST cluster types (CT), and allelic profiles of the 1,861 cgMLST genes (23) were reported to A.M., the ring trial organizer.

**TABLE 2:** Characteristics of the 20 human S. aureus isolates that were sent as DNA samples to the five participating laboratories in a blinded fashion and used as controls

Sample ID		Spa type (based on Sanger sequencing)	Comment/reference
Ring trial	Original		
NGSRT01	00468	t011	Livestock-associated MRSA
NGSRT02	00551	t011	Livestock-associated MRSA, identical cgMLST genotype as NGSRT01
NGSRT03	01346	t011	Livestock-associated MRSA
NGSRT04	01354	t010	Classical hospital-acquired MRSA
NGSRT05	01360	t011	Livestock-associated MRSA, identical cgMLST genotype as NGSRT03
NGSRT06^*a*^	02180	t002	Central European community-acquired PVL^*b*^-positive MRSA
NGSRT07^*a*^	02482	t008	US typical community-acquired PVL-positive MRSA
NGSRT08^*a*^	02560	t044	Central European community-acquired PVL-positive MRSA
NGSRT09^*a*^	02638	t012	Classical hospital-acquired MRSA
NGSRT10^*a*^	02786	t843	*mecC*-positive MRSA
NGSRT11^*a*^	02949	t843	*mecC*-positive MRSA
NGSRT12^*a*^	02994	t003	Classical hospital-acquired MRSA
NGSRT13^*a*^	03039	t032	Classical hospital-acquired MRSA
NGSRT14^*a*^	COL	t008	MRSA strain COL
NGSRT15^*a*^	COL	t008	Duplicate of MRSA reference strain COL
NGSRT16	ATCC 25923	t021	MSSA quality control strain ATCC 25923
NGSRT17	P1	t001	Isolate P1 from reference 23
NGSRT18	P3	t001	Isolate P3 from reference 23
NGSRT19	P4	t001	Isolate P4 from reference 23, identical cgMLST genotype as NGSRT18
NGSRT20	P12	t001	Isolate P12 from reference 23

a These samples were separately cultivated, and DNA was extracted and sequenced as controls.

b PVL, Panton-Valentine leukocidin.

In parallel, ST and rST were also determined from the assembly contigs using the BIGSdb system (21, 22, 24). Moreover, spa typing data using Sanger sequencing were available for all isolates (Table 2). Furthermore, 10 strains (NGSRT06 to NGSRT15; in the following-named control) were separately cultivated, and DNA was extracted and sequenced in the ring trial organizer's laboratory. For detailed analysis and visualization, a minimum-spanning tree based on the reported cgMLST allelic profiles was constructed using SeqSphere^+^ with the option “pairwise ignoring missing values” turned on. Finally, as we had included as NGSRT16 the well-known quality control strain ATCC 25923 that was recently completely sequenced (12), we determined whether potential discrepancies were due to NGS sequencing errors during the ring trial. Discrepancies that were detected between the published sequence (NZ_CP009361) and the ring trial data from all participants were further analyzed by bidirectional Sanger sequencing from the same DNA that was also sent to the participants. For further confirmatory Sanger sequencing, flanking regions of approximately 250 nucleotides up- and downstream of the detected discrepancies were extracted from the genome sequence, and primers were designed using the NCBI Primer-BLAST service (25). The amplified fragments were purified and Sanger sequenced as described previously (26). The resulting chromatogram files were also analyzed using the SeqSphere^+^ software.

### Accession number(s).

Raw reads are deposited at European Nucleotide Archive (ENA) under study accession number PRJEB15231.

## Supplementary Material

Supplemental material
