# Supplementary material for: High Interlaboratory Reproducibility and Accuracy of Next-Generation-Sequencing-Based Bacterial Genotyping in a Ring Trial
Source: J Clin Microbiol. 2017 Feb 22;55(3):908–13. doi: 10.1128/JCM.02242-16 (PMC5328459; doi:10.1128/JCM.02242-16)
Supplement: Supplemental material [file supp_55_3_908__index.html]

High Interlaboratory Reproducibility and Accuracy of Next-Generation-Sequencing-Based Bacterial Genotyping in a Ring Trial — Supplemental material 

# High Interlaboratory Reproducibility and Accuracy of Next-Generation-Sequencing-Based Bacterial Genotyping in a Ring Trial

## Supplemental material

- Supplemental file 1 -

  Table S1 (Ring trial results)

  XLSX, 852K
- Supplemental file 2 -

  Table S2 (List of oligonucleotides used for amplication and sequencing of discrepant single nucleotide variants in *S. aureus* strain ATCC 25923 in comparison to the ring trial whole-genome sequencing data for the 1,861 cgMLST target genes)

  XLSX, 16K
- Supplemental file 3 -

  Ring trial instructions

  PDF, 361K
