# Supplementary material for: High Interlaboratory Reproducibility and Accuracy of Next-Generation-Sequencing-Based Bacterial Genotyping in a Ring Trial
Source: J Clin Microbiol. 2017 Feb 22;55(3):908–13. doi: 10.1128/JCM.02242-16 (PMC5328459; doi:10.1128/JCM.02242-16)
Supplement: Supplemental material [file JCM.02242-16_zjm999095395s3.pdf]

Instructions for *S. aureus* Ring-Trial With SeqSphere+ Analysis

Preliminaries

1. Make sure that you have the latest **SeqSphere+ version 2.4.1** or later installed, or later. The version can be checked by invoking the menu “Help | About Ridom SeqSphere+”.

2. We would like to ask you to perform **one MiSeq Nextera XT 250bp paired-end run** (v2 chemistry; run time about 40h) with the supplied 20 DNA samples. To ensure that we have enough coverage for high-quality analysis, we would like to ask to put no other samples onto this run. Make sure that your **run** meets the **minimum specifications** with respect to **output** (i.e., **5.6 Gb** that for an average unassembled and unprocessed coverage of at least 100-fold for the 20 samples) and **>Q30-Score** (i.e., **>75%** following the Illumina specifications). You find this information by browsing with the **Illumina Sequence Analysis Viewer** (SAV) software to the run output folder that contains the InterOp subdirectory. If the **minimum specifications were not met, please repeat the run**. To document your run please open in SAV the “Summary” tab and click on the “Copy to Clipboard …” button (see Figure 1). Open MS Excel, paste the clipboard content into an empty sheet by preserving the formatting and save it with a file name containing your according laboratory ID (e.g., C1_InterOp.xlsx).

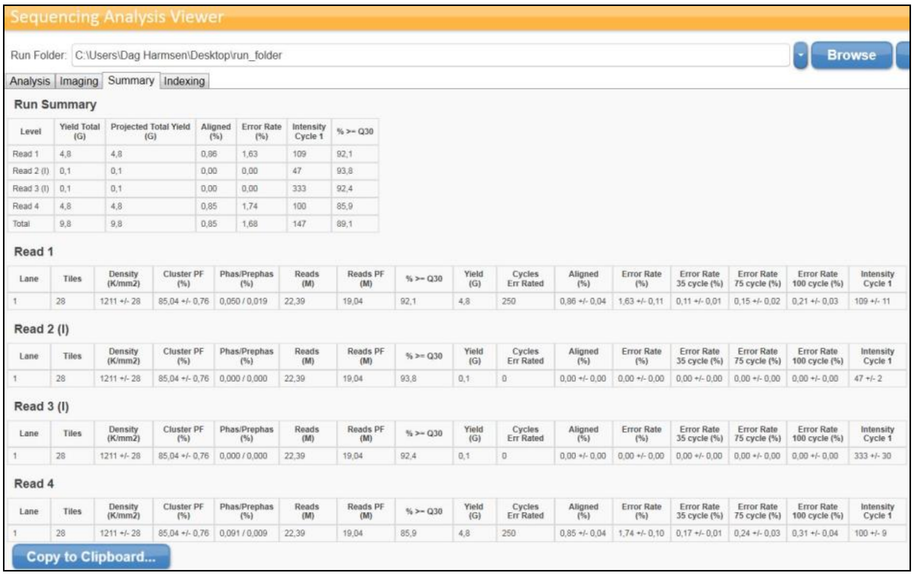

Figure 1: Example of SAV Summary

3. Make sure that the FASTQ files are stored in a directory accessible from the computer where the SeqSphere+ client is installed. The names of the FASTQ files must start with the **Ring-trial Sample ID** of the strain as sent to you by Alexander Mellmann (e.g., NGSRT01C1 for the first strain of laboratory C1). The Illumina software will add some further information to the file name (e.g., NGSRT01C1_S2_L001_R1_001.fastq.gz).

Define Project and Check Nomenclature Server Registration Status

1. Start SeqSphere+ and login with your account.
2. Invoke in the menu “File | New | Project” to start a new project
3. Enter the Project name, e.g., “NGS Ring-trial”, in the upcoming dialog

4. Press the button “Add from Store” and choose *Staphylococcus aureus* in the upcoming dialog
5. Press the button “Select All” to select the four Task Templates that are available for *S. aureus* (S. aureus cgMLST, S. aureus Accessory, S. aureus MLST, and S. aureus spa typing). Press OK to add the Task Templates to your Project.
6. Download from ftp://128.176.45.182/* the file “rMLST.tasktemplate”**.
7. Press the button “Add Manually”. In the upcoming dialog press the button “Import from File” and choose the downloaded file “rMLST.tasktemplate”. Press the “OK” button in the “Import Task Template” dialog window to confirm the import. Press OK to add the imported Task Template to your Project.
8. Press OK to save the Project.
9. Invoke in the menu “Options | User Settings”. Click on the tab “Nomenclature Server Account” in the upcoming dialog. If your account data is shown here, you are already registered on the Nomenclature Server, and you can close the dialog and continue with the next step. Else, if you only see the message “No Nomenclature Server user registered!”, then press the button “Register New Account” on the bottom of the panel, and enter your contact details in the upcoming dialog. Press the button “Register” to confirm and close the dialog.
10. Invoke in the menu ”File | Logout”

*same account as used for uploading your FASTQ files.
**the template is only meant for this academic ring-trial purpose, please delete afterwards. If you intend to use rMLST permanently, please register at and use the University Oxford rMLST service (http://pubmlst.org/rmlst/).

Define and Start Pipeline Script

1. Invoke in the menu “File | Start Pipeline Mode”
2. Press the button “Create New Script” in the upcoming Pipeline Mode dialog
3. Login with your user name and password and press the button “Next” to continue with “Define Script Name and Comment”
4. Enter a Pipeline Name, e.g., “NGS Ring-trial”, and press the button “Next” to continue with “Define Input Sources”
5. Press the button Choose at the File Location field and select the directory of the FASTQ files. Choose “Illumina” in the Sequencing Platform field. The Ring-trial Sample ID should then be highlighted in blue in the File preview panel (see Figure 2). Press the button “Next” to continue with “Define Projects”.

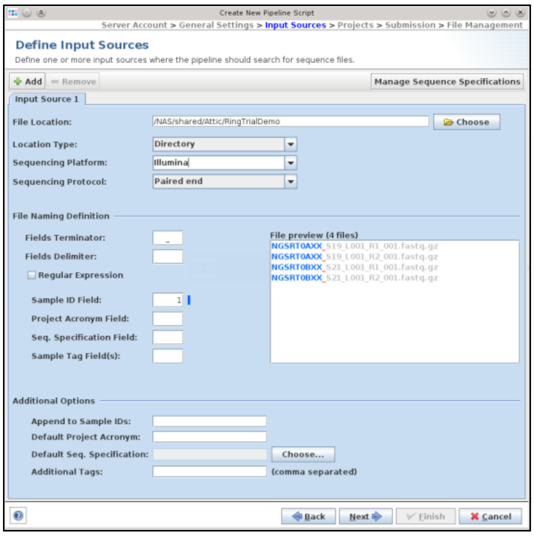

Figure 2: Define Pipeline Input Source

6. Choose in the field Project Name the previously defined “NGS Ring-trial” project. Check the box “Perform Assembling/Mapping for read files”. Press the button “Next “to continue with “Define Submission”.
7. Check the box “Automatically submit alleles and Sample data to Nomenclature Server”. Choose “Submit” in the field Submit Sample ID. As no place and time information was delivered by Alexander choose “Do not submit (use 'unknown')” in the two fields “Submit from source location” and “Submit from collection data”. Check the privacy box at the bottom of the dialog (see Figure 3). Press the button “Next” to continue with “Define File Management”.

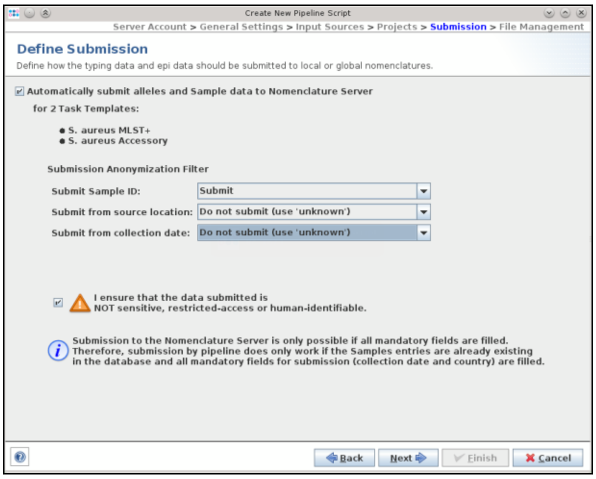

Figure 3: Define Pipeline Submission

8. Select “Upload files to SeqSphere^{+} Server” in the section “Assembled/Mapped Files (ACE/BAM). Press the button “Test Pipeline Script” at the bottom of the dialog. The just defined script will be tested and you should receive within a few seconds the message “Pipeline test succeeded”. Press the button “OK” to close the message dialog. Press the button “Finish” to store your pipeline script.
9. Press the button “Start Script” in the upcoming window to start your just defined pipeline script. If you use a computer equipped with the minimum recommended amount of RAM (i.e., >16 GB) for SeqSphere^{+}, the analysis of the 20 samples will take about 7 hours. You could speed- and scale-up the analysis by executing the pipeline script on other computers concurrently. Once the analysis is finished press the button “Exit”.

Export the Analysis Results

1. Start SeqSphere$^+$ and login with your account. Invoke in the menu Tools | Comparison Table.
2. Press the button “New Definition” in the upcoming dialog. Choose the previously defined “NGS Ring-trial” project in the Project field. In the section “Query Results” select cgMLST, MLST (both are selected by default), rMLST, and spa-typing. Do not use Accessory! Check the box “Store Definition” and add your laboratory ID to the suggested “Name” NGS Ring-trial (e.g., NGS Ring-trial_C1). Press the button “Choose Sample Fields”. In the upcoming dialog scroll down to “Sequence Specifications” and check the box to select all fields below (see Figure 4). Press the button “OK” to close the “Choose Sample Fields” dialog. Press again the button “OK” to store the Comparison Table Definition for later reuse and to open the table immediately.

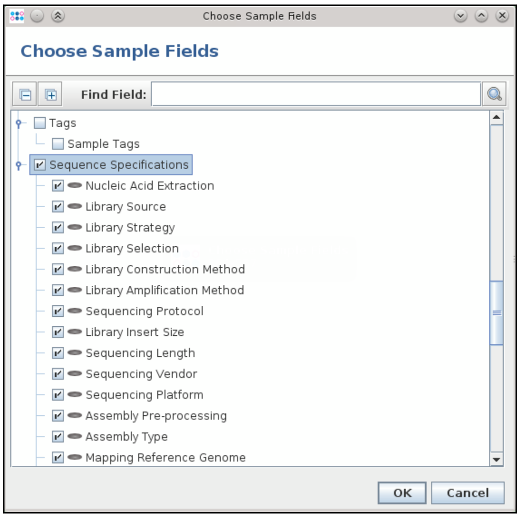

Figure 4: Choose Sample Fields

3. Invoke in the upcoming Comparison Table window the menu “File | Export Table Data” and save the table with the suggested file name (e.g., NGS Ring-trial_C1.xlsx).

Send the Analysis Results to Münster

1. Send by **email** to Alexander Mellmann (Alexander.Mellmann@ukmuenster.de) the exported Comparison Table (e.g., NGS Ring-trial_C1.xlsx) and the run document (e.g., C1_InterOp.xlsx).
2. To be able to submit the FASTQs of all participants in one ENA study we ask you also to **upload** your FASTQs to the **Münster FTP Server**. Please upload your data into the according subdirectories (e.g., C1). The FTP Server address is as following: ftp://128.176.45.182 (usual ftp port 21; not sftp port 22). The account name and password will be supplied in a separate email immediately following the email containing this document attached.
3. Please keep also locally your FASTQ files and the InterOp subdirectory for possible later usage.

Document version 1.1; June 22th, 2015
